# The validity and reliability of a digital Ruff Figural Fluency Test (RFFT)

**DOI:** 10.1186/s40359-021-00566-x

**Published:** 2021-04-28

**Authors:** J. Vrijsen, C. L. van Erpecum, S. E. de Rooij, J. Niebuur, N. Smidt

**Affiliations:** 1 University Medical Centre Groningen, Department of Epidemiology, University of Groningen, Hanzeplein 1, FA40, P.O. Box 30 001, 9700 RB Groningen, The Netherlands; 2 University Medical Center Groningen, Department of Internal Medicine, University of Groningen, Groningen, The Netherlands

**Keywords:** Cognition, Cognitive dysfunction, Executive function, Neuropsychological test, Ruff Figural Fluency Test, Software, Reproducibility of results, Validation study

## Abstract

**Background:**

The Ruff Figural Fluency Test (RFFT) is a valid but time-consuming and labour-intensive cognitive paper-and-pencil test. A digital RFFT was developed that can be conducted independently using an iPad and Apple Pencil and RFFT scores are computed automatically. We investigated the validity and reliability of this digital RFFT.

**Methods:**

We randomly allocated participants to the digital or paper-and-pencil RFFT. After the first test, the other test was performed immediately (cross-over). Participants were invited for a second digital RFFT 1 week later. For the digital RFFT, an (automatic) algorithm and two independent raters (criterion standard) assessed the number of unique designs (UD) and perseverative errors (PE). These raters also assessed the paper-and-pencil RFFT. We used Intraclass correlation coefficients (ICC), sensitivity, specificity, %-agreement, Kappa, and Bland–Altman plots.

**Results:**

We included 94 participants (mean (SD) age 39.9 (14.8), 73.4% follow-up). Mean (SD) UD and median (IQR) PE of the digital RFFT were 84.2 (26.0) and 4 (2–7.3), respectively. Agreement between manual and automatic scoring of the digital RFFT was high for UD (ICC = 0.99, 95% CI 0.98, 0.99, sensitivity = 0.98; specificity = 0.96) and PE (ICC = 0.99, 95% CI 0.98, 0.99; sensitivity = 0.90, specificity = 1.00), indicating excellent criterion validity. Small but significant differences in UD were found between the automatic and manual scoring (mean difference: − 1.12, 95% CI − 1.92, − 0.33). Digital and paper-and-pencil RFFT had moderate agreement for UD (ICC = 0.73, 95% CI 0.34, 0.87) and poor agreement for PE (ICC = 0.47, 95% CI 0.30, 0.62). Participants had fewer UD on the digital than paper-and-pencil RFFT (mean difference: − 7.09, 95% CI − 11.80, − 2.38). The number of UD on the digital RFFT was associated with higher education (Spearman’s *r* = 0.43, *p* < 0.001), and younger age (Pearson’s *r* = − 0.36, *p* < 0.001), showing its ability to discriminate between different age categories and levels of education. Test–retest reliability was moderate (ICC = 0.74, 95% CI 0.61, 0.83).

**Conclusions:**

The automatic scoring of the digital RFFT has good criterion and convergent validity. There was low agreement between the digital RFFT and paper-and-pencil RFFT and moderate test–retest reliability, which can be explained by learning effects. The digital RFFT is a valid and reliable instrument to measure executive cognitive function among the general population and is a feasible alternative to the paper-and-pencil RFFT in large-scale studies. However, its scores cannot be used interchangeably with the paper-and-pencil RFFT scores.

**Supplementary Information:**

The online version contains supplementary material available at 10.1186/s40359-021-00566-x.

## Background

Cognitive decline can be a normal part of ageing [1]. In some people, it can accelerate, ultimately leading to mild cognitive impairment or dementia [2]. Pathological cognitive decline is a long-term neurodegenerative process that begins approximately 10–20 years before dementia is clinically diagnosed [3–5]. To get insight into the aetiology of dementia and in potential effects of preventive efforts, cognitive functioning should be assessed within large-scale longitudinal cohort studies with a long follow-up period.

One of the first signs of cognitive decline is a decline of executive functioning, which is used to control and coordinate cognitive tasks and behaviour [6]. Within the domain of executive functioning, one can distinguish between the verbal fluency domain and the non-verbal fluency domain. Tests assessing non-verbal fluency are more sensitive in detecting changes in executive functioning throughout the life course and should therefore be preferred over verbal fluency tests [7, 8].

The Ruff Figural Fluency Test (RFFT) is a paper-and-pencil test assessing non-verbal fluency, which tends to have better sensitivity in detecting early changes in non-verbal fluency [7]. The RFFT consists of an assignment in which respondents are instructed by a trained examiner to draw as many unique designs as possible on a sheet of 35 boxes within 60 s. This task is repeated five times, each time using a sheet containing different point configurations [9]. The performance on the RFFT is assessed by counting the total number of unique designs and the total number of perseverative errors (i.e., double designs) from these five sheets. Previous research showed that the RFFT has a good construct validity [10], and can discriminate adequately between different groups of educational level and age [9, 11, 12].

However, the utility of the RFFT is limited because its administration and scoring are a time-consuming and labour-intensive task. More specifically, for administration, a trained examiner is needed to provide instructions for each sheet and conduct of the subsequent assessment [13]. For scorings, a trained rater is needed to evaluate examinees’ performances according to the manual. Accordingly, the feasibility of the RFFT is undermined particularly in large-scale cohort studies. To resolve these limitations, Elderson et al. [13] developed an automatic pattern recognition algorithm to evaluate examinees’ performances on the RFFT. The algorithm showed high agreement with those evaluated by human raters and thus, improves the feasibility [13]. However, the algorithm cannot completely resolve the aforementioned limitations, because it still needs human raters to provide instructions and conduct subsequent assessment. Moreover, the RFFT sheets should be scanned manually, before the algorithm works. Thus, the labour-intensive administration remains a limitation of the RFFT and interferes with its application.

To resolve all RFFT limitations simultaneously, a digital version of the RFFT was developed. The digital RFFT can be performed independently on an iPad Pro (2018) with an Apple Pencil (2nd generation) and headphone. The digital RFFT has at least three advantages. First, the administration and scoring can be conducted automatically and thus, it requires no rater training and can release heavy burdens on human raters. Second, uniform instructions are provided by the iPad and therefore, reduce the variability caused by inter-rater differences. Third, the RFFT scores can be provided and used directly for result interpretations and further data analyses. Thus, the digital RFFT shows great feasibility in large-scale cohort studies. However, the utility of the digital RFFT is constrained due to the unknown psychometric properties in the target population. The objectives of this study were to validate the newly developed digital RFFT, firstly in terms of *criterion validity* and *convergent validity*, and secondly in terms of *test–retest reliability*, among adults from the Dutch general population.

## Methods

### Study design

The study consisted of two visits. During the first visit (cross-sectional validity study design), participants were randomly allocated to either the digital RFFT or the paper-and-pencil RFFT using block randomization (block size of four) stratified for gender, age group (< 40 years, 40–59 years, or ≥ 60 years), and highest level of completed education (low, middle, or high, based on the International Standard Classification of Education [14] (Additional file 1: Appendix 1)). We used a random number generator for the randomization. After the first test (digital RFFT or paper-and-pencil RFFT), the other test was performed (cross-over). Participants were invited to return for a second visit 1 week after the first visit, in which only the digital RFFT was repeated (test–retest reliability study design). For this study, ethical approval was obtained by the medical ethical committee of the University Medical Centre Groningen (trial number METc 2019/389, date of approval 23/07/2019). This research was carried out in accordance with relevant guidelines and regulations.

### Study population

Participants were recruited during a 6-week period in July and August 2019 through posters and flyers, convenience sampling and online advertising. Individuals interested in participation could make an appointment by using an online registration website (or by telephone) for a first and second visit (after 1 week) at the research site. Afterwards, participants received a voucher of 10 euros as an incentive to participate. Participants were deemed eligible if they (1) were 18 years or older, (2) provided written informed consent, (3) understand the Dutch language, and (4) did not have impairments in writing with the dominant hand, hearing, or vision.

### Data collection

#### Digital RFFT (first and second visit)

Participants performed the digital RFFT independently within an application using an Apple iPad Pro (2018, 12.9 inch, 64 GB), an Apple Pencil (2nd generation), and headphone. The software for the application was developed by Bruna & Bruna (www.brunabruna.nl). The digital RFFT started with a video instruction about the assignment. In line with the Standard Operating Procedure of the RFFT [15], participants also received feedback on the performance of the practice sheets through correction videos on the iPad. If instructions were not clear enough yet, participants were also able to watch example videos before and during the tests showing both simple and more complex examples for each point configuration.

#### Paper-and-pencil RFFT (first visit only)

**Fig. 1:**
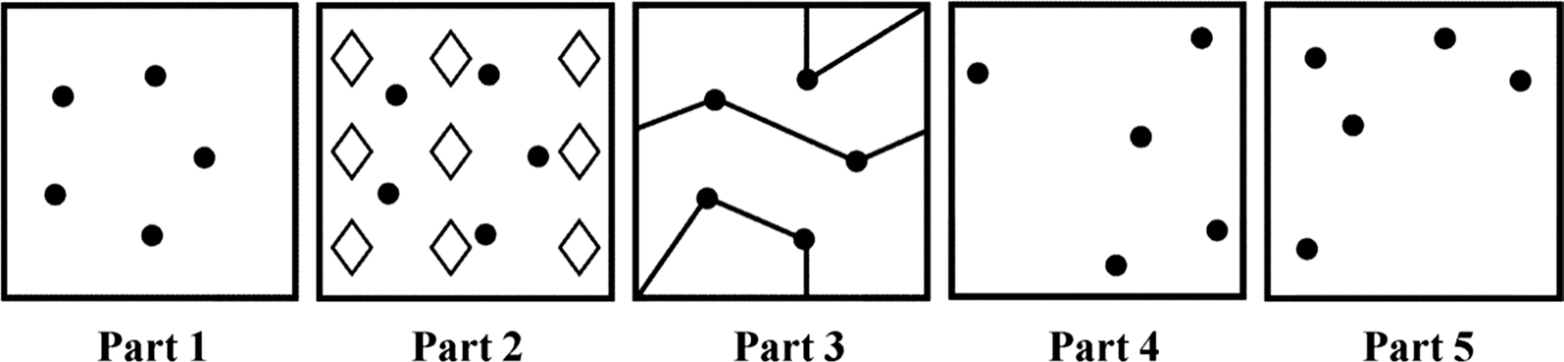
The five RFFT sheets [9]

During the paper-and-pencil RFFT, a trained examiner provided test instructions according to the Standardized Operating Procedure of the RFFT [15]. First, participants received a practice sheet with three boxes on which they could draw unique designs by connecting two or more dots. The trained examiners corrected the participant if needed. Then, the participants performed this task on a sheet of 35 boxes with identical configurations of points, in which they should draw as many unique designs as possible within 60 s. The participants performed these tasks on a total of five different practice and test sheets which consisted of different point configurations (Fig. 1).

The paper-and-pencil RFFT was performed on an 8.5 × 11″ sheet of paper with a red marker. All five RFFT sheets have a different point configuration.

#### Scoring of the RFFT sheets

For the digital RFFT, each individual box was automatically identified as a unique design, perseverative error, erroneous design, or empty box through an algorithm. Criteria for identifying unique designs, perseverative errors, erroneous designs and empty boxes are shown in Additional file 1: Appendix 2. Subsequently, the number of unique designs and perseverative errors were automatically computed and stored in a database.

For the digital and paper-and-pencil RFFT at the first visit, two independent and trained human raters identified each individual box as a unique design, perseverative error, erroneous design, or empty box. Furthermore, they scored the number of unique designs and perseverative errors. Additional scoring was performed when the two raters’ number of unique designs or perseverative errors differed on more than two points in one sheet or more than four points on the total score of the five sheets [13]. Subsequently, agreement by the two raters was obtained through a consensus meeting. If the two raters’ number of unique designs or perseverative errors differed two points or less in one sheet or four points or less on the total score for the five sheets, the scores of the two raters were averaged. The fact that scoring of the digital RFFT at the first visit was also performed by human raters allowed us to compare the automatic and manual scoring of the digital RFFT, and thereby, to evaluate the scoring performance of the algorithm against a common reference standard.

#### Questionnaire

Participants filled out a questionnaire on the socio-demographic characteristics age, gender, and highest level of completed education. Highest level of completed education was categorized into low, middle, and high based on the International Standard Classification of Education [14] (Additional file 1: Appendix 1). Additionally, highest level of education was also dichotomized into ≤ 12 years of education and > 12 years of education [16]. Furthermore, for practicability purposes, the trained examiner reported potential problems of the digital RFFT as well as how often the participants watched the videos with examples.

### Statistical methods

Descriptive statistics were provided for the entire study population, and separately for the two randomized groups (i.e. group that started with the digital RFFT and the group that started with the paper-and-pencil RFFT). Differences in demographic characteristics and the number of unique designs and perseverative errors of the RFFT (digital, paper-and-pencil) between the randomized groups were assessed using two-sample t-test (normally distributed continuous variables), Mann–Whitney U test (non-normally distributed continuous variables), and a Chi-Square test (categorical variables).

We examined the *criterion validity* of the digital RFFT from two perspectives. First, we examined the congruence between the scores provided by the digital RFFT and those from human raters (gold standard). Specifically, the number of unique designs and perseverative errors were compared between the automatic and manual scorings. For this purpose, we computed the intraclass correlation coefficient (ICC; absolute, two-way mixed), a Lin’s Concordance Correlation Coefficient (LCCC) for replacement testing, and a Bland–Altman plot. Moreover, to further examine whether the automatic scorings of digital RFFT can correctly identify individual boxes as unique designs and perseverative errors, we calculated the sensitivity and specificity using the manual scorings as the reference standard. Second, we examined the congruence between the scores provided by the digital RFFT and those from paper-and-pencil RFFT. For this purpose, we computed the ICC (absolute, two-way mixed) and Bland–Altman plots.

Secondly, we investigated *convergent validity*, which refers to the congruence between the digital RFFT and theoretically related constructs [17]. Specifically, we examined the correlation between the number of unique designs and perseverative errors of the digital RFFT during the first visit (automatic scoring) with age and education level. For this purpose, we used Pearson’s correlation coefficient for the normally distributed variables and Spearman’s correlation coefficient for non-normally distributed variables.

Thirdly, we investigated *test–retest reliability* of the digital RFFT, which refers to the congruence between test scores on different occasions, assuming that the participant’s ability remains the same [17]. For this purpose, we compared the number of unique designs and perseverative errors between the first and second visit based on the automatic scoring. Here, we provided an ICC (absolute, two-way mixed) and a Bland–Altman plot.

For criterion validity, convergent validity, and test–retest reliability, we considered ICC values below 0.50, between 0.50 and 0.74, between 0.75 and 0.90, and above 0.90 as poor, moderate, good, and excellent, respectively [18].

## Results

A total of 94 individuals aged between 18 and 76 years participated in the study and performed both the digital RFFT and the paper-and-pencil RFFT at the first visit. Afterwards, 69 participants (73.4% of the eligible participants) also performed the digital RFFT during the second visit. Participants from the first visit had a mean (SD) age of 39.9 (14.8) years. More than half of these participants was female (58.5%), and 74.5% of the participants was highly educated (Table 1).

**Table 1:** Characteristics of the study population

	Total study population (N = 94)	Allocation		*P* value
		Digital RFFT first (N = 50)	Paper-and-pencil RFFT first (N = 44)	
Sex (female)	*55* (58.5)	*27* (54.0)	*28* (63.6)	0.66
Age in years, mean (SD)	39.9 (14.8)	41.3 (15.5)	38.4 (13.8)	0.29
Age categories				0.87
< 40 years	*55* (58.5)	*28* (56.0)	*27* (61.4)	
40–59 years	*25* (26.6)	*14* (28.0)	*11* (25.0)	
≥ 60 years	*14* (14.9)	*8* (16.0)	*6* (13.6)	
Educational level				0.75
Low	*11* (11.7)	*7* (14.0)	*4* (9.1)	
Middle	*13* (13.8)	*7* (14.0)	*6* (13.6)	
High	*70* (74.5)	*36* (72.0)	*34* (77.3)	
Years of education				0.32
≤ 12 years education	*26* (27.7)	*16* (32.0)	*10* (22.7)	
> 12 years education	*68* (72.3)	*34* (68.0)	*34* (77.3)	

*N* (%) is presented unless indicated otherwise

*SD* standard deviation, *IQR* interquartile range

**Fig. 2:**
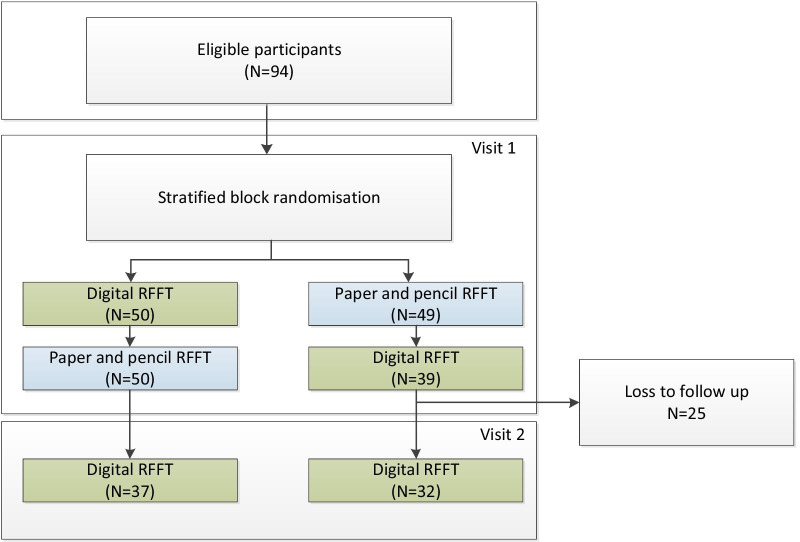
Flowchart of inclusion of participants

Overall, 50 participants were allocated to the group that first received the digital RFFT and 44 participants were allocated to the group that first received the paper-and-pencil RFFT (Fig. 2). We detected small but statistically non-significant differences between participants starting with the digital RFFT and participants starting with the paper-and-pencil RFFT. Compared to participants starting with the digital RFFT, participants starting with the paper-and-pencil RFFT were slightly younger (mean (SD) age: 38.4 (13.8) versus 41.3 (15.5) and more often highly educated (77.3% versus 72.0%). The median (IQR) duration of the digital RFFT and the paper-and-pencil RFFT was 12 min (11–14) and 10 min (9–11), respectively.

Participants starting with the digital RFFT had a mean (SD) of 74.1 (24.4) unique designs and a median (IQR) of 4 (2–7.3) perseverative errors on the digital RFFT based on the automatic scoring. When these participants subsequently performed the paper-and-pencil RFFT, they had a mean (SD) of 96.9 (22.5) unique designs and a median (IQR) of 5 (2–8.5) perseverative errors (Table 2).

**Table 2:** RFFT scores for the study population

	Total study population (N = 94)	Allocation		*P* value
		Digital RFFT first (N = 50)	Paper-and-pencil RFFT first (N = 44)	
First visit				
Digital RFFT				
UD (automatic), mean(sd)	84.2 (26.0)	74.1 (24.4)	96.5 (23.3)	< 0.01
PE (automatic), median (IQR)	4 (2–7.3)	4 (2–7.3)	4.5 (2.25–7.8)	0.74
UD (manual), mean(sd)	85.3 (26.2)	75.1 (25.2)	96.5 (23.2)	< 0.01
PE (manual), median (IQR)	4.5 (2–7.5)	4.75 (2–7.5)	4 (2.5–7.4)	0.99
Duration in minutes, median (IQR)	12 (11–14)	13 (12–14)	12 (12–12.8)	< 0.01
Paper-and-pencil RFFT				
UD (manual), mean (sd)	91.3 (22.7)	96.9 (22.5)	85.3 (20.8)	0.02
PE (manual), median (IQR)	4.5 (2–8)	5 (2–8.5)	4 (2–7)	0.29
Duration in minutes, median (IQR)	10 (9–11)	9 (9–10)	11 (10–12)	< 0.01
Second visit				
Digital RFFT				
UD (automatic), mean (SD)	104.4 (22.7)	102.5 (22.4)	106.6 (23.2)	0.46
PE (automatic), median (IQR)	6 (2–8.3)	6 (2–7.5)	6 (4–9)	0.57

*N* (%) is presented unless indicated otherwise

*UD* unique designs, *PE* perseverative errors, *SD* standard deviation, *IQR* interquartile range

Participants starting with the paper-and-pencil RFFT had a mean (SD) of 85.3 (20.8) unique designs and a median (IQR) of 4 (2–7) perseverative errors on the paper-and-pencil RFFT. When these participants subsequently performed the digital RFFT, they had a mean (SD) of 96.5 (23.3) unique designs and a median (IQR) of 4.5 (2.3–7.8) perseverative errors based on the automatic scoring.

### Criterion validity: comparison between automatic and manual scoring of the digital RFFT

**Fig. 3:**
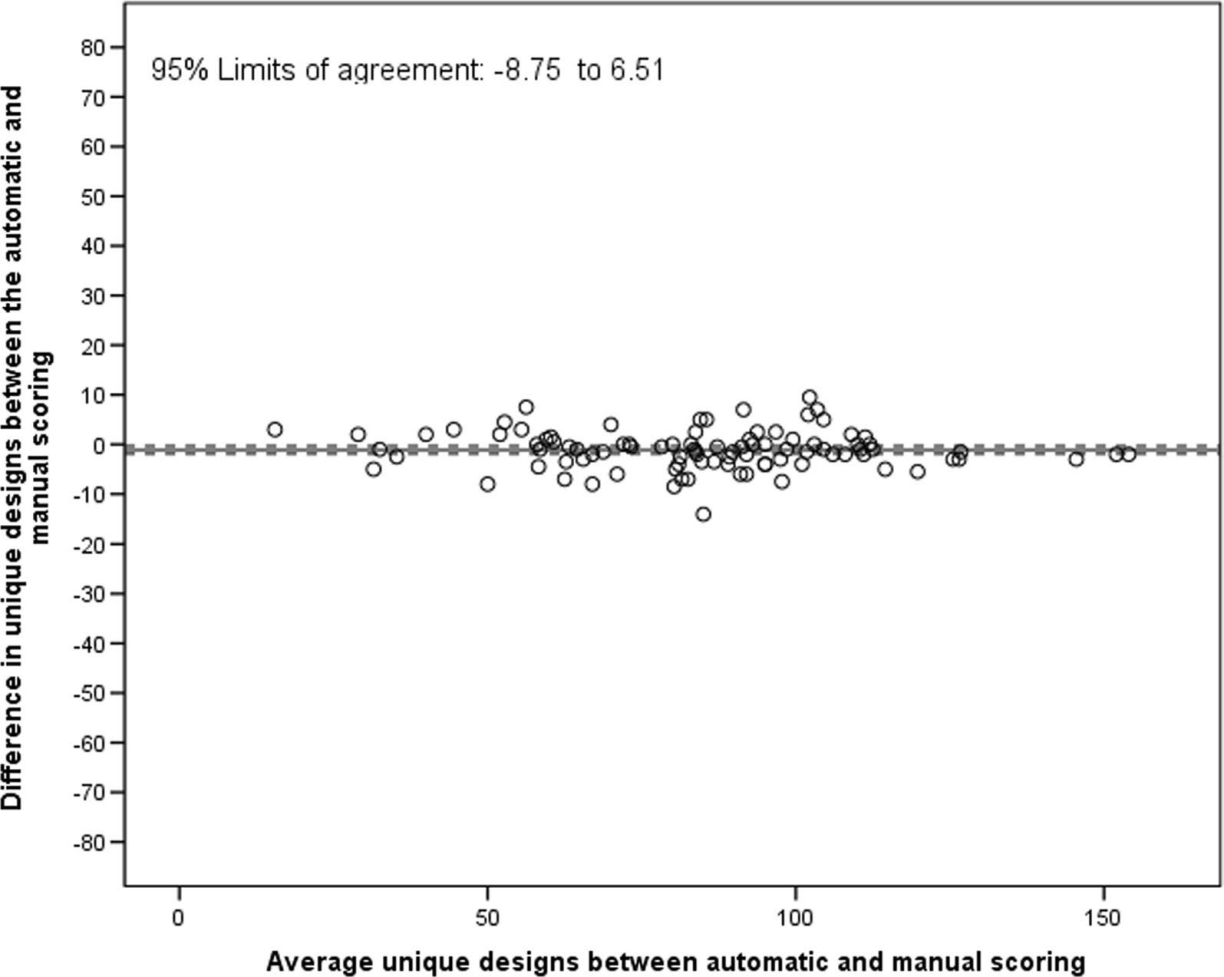
Bland–Altman plot of automatic and manual scoring of the digital RFFT (number of unique designs)

For the number of unique designs, the ICC and LCCC between the automatic and manual scoring of the digital RFFT were 0.99 (95% CI 0.98, 0.99) and 0.99 (95% CI 0.98, 0.99), respectively. However, the number of unique designs assessed by automatic scoring was significantly smaller than those assessed by manual scoring of the digital RFFT (mean difference = − 1.12 (95% CI − 1.92, − 0.33; Fig. 3). This systematic difference did not get more pronounced with a higher average number of unique designs on the automatic and manual scoring. The 95% limits of agreement were − 8.75 and 6.51. For detecting an individual box as a unique design, the automatic scoring had a sensitivity of 0.98 and a specificity of 0.96.

**Fig. 4:**
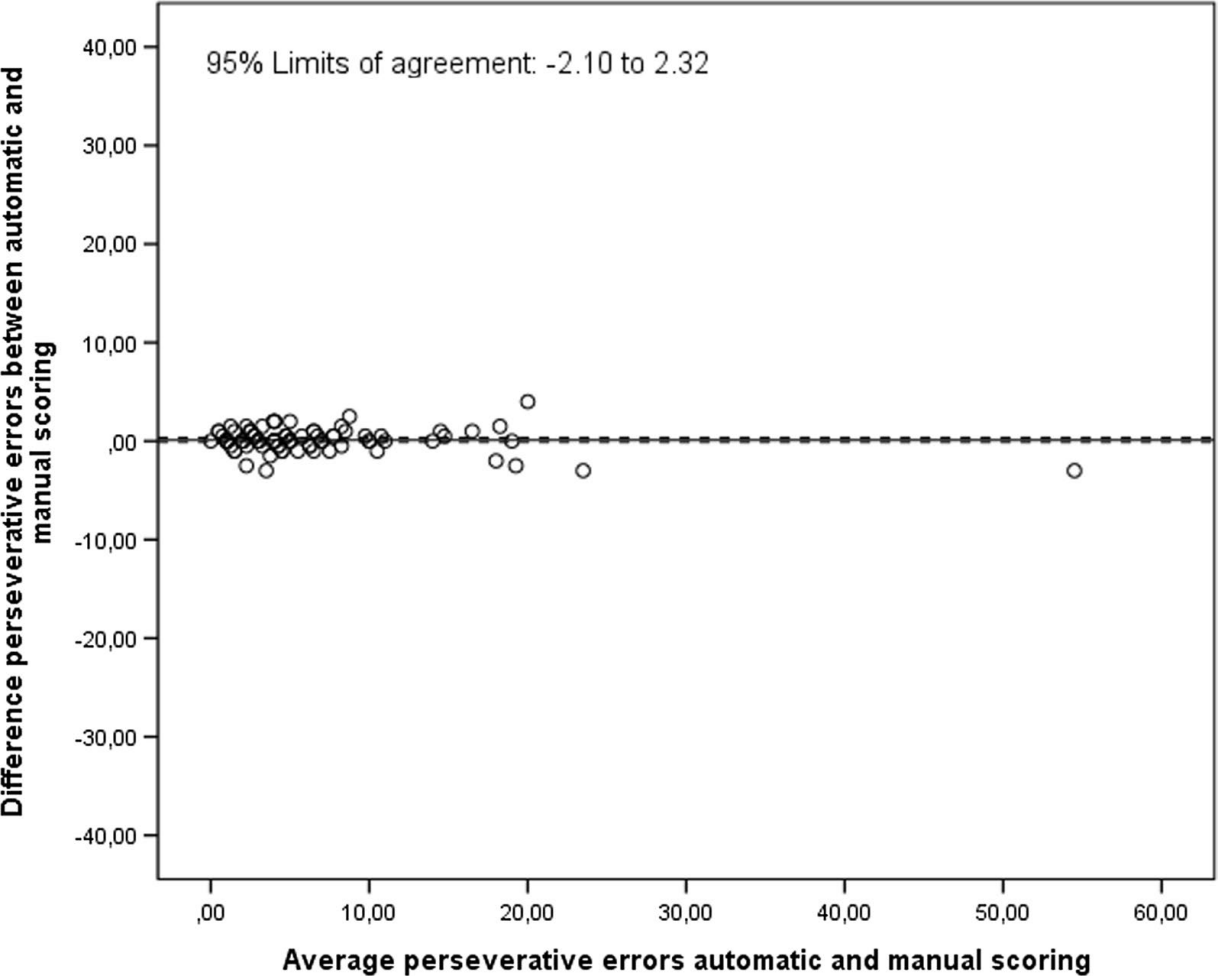
Bland–Altman plot of automatic and manual scoring of the digital RFFT on the number of perseverative errors

For the number of perseverative errors, the ICC and LCCC between automatic and manual scoring of the digital RFFT were 0.99 (95% CI 0.98, 0.99) and 0.99 (95% CI 0.98, 0.99), respectively. The number of perseverative errors on the digital RFFT with automatic scoring did not differ significantly from those of the digital RFFT with manual scoring (mean difference = 0.11 (95% CI − 0.12, 0.34; Fig. 4). The 95% limits of agreement were − 2.10 and 2.32. For detecting an individual box as a perseverative error, the automatic scoring had a sensitivity of 0.90 and a specificity of 1.00.

Overall, there was good agreement among the two human raters for the digital RFFT (percentage agreement = 94; weighted Kappa = 0.90). Besides, they had good agreement on the number of unique designs and perseverative errors for the digital RFFT (ICC = 0.98, 95% CI: 0.96, 0.99; and ICC = 0.98, 95% CI 0.97, 0.98, respectively). For the paper-and-pencil RFFT, the two human raters also had high agreement (percentage agreement = 93, weighted Kappa = 0.87). Furthermore, they had excellent agreement on the number of unique designs and perseverative errors on the paper-and-pencil RFFT (ICC = 0.94, 95% CI 0.87, 0.97; ICC = 0.84, 95% CI: 0.71, 0.90, respectively).

### Criterion validity: comparison between the digital RFFT (automatic scoring) and paper-and-pencil RFFT

**Fig. 5:**
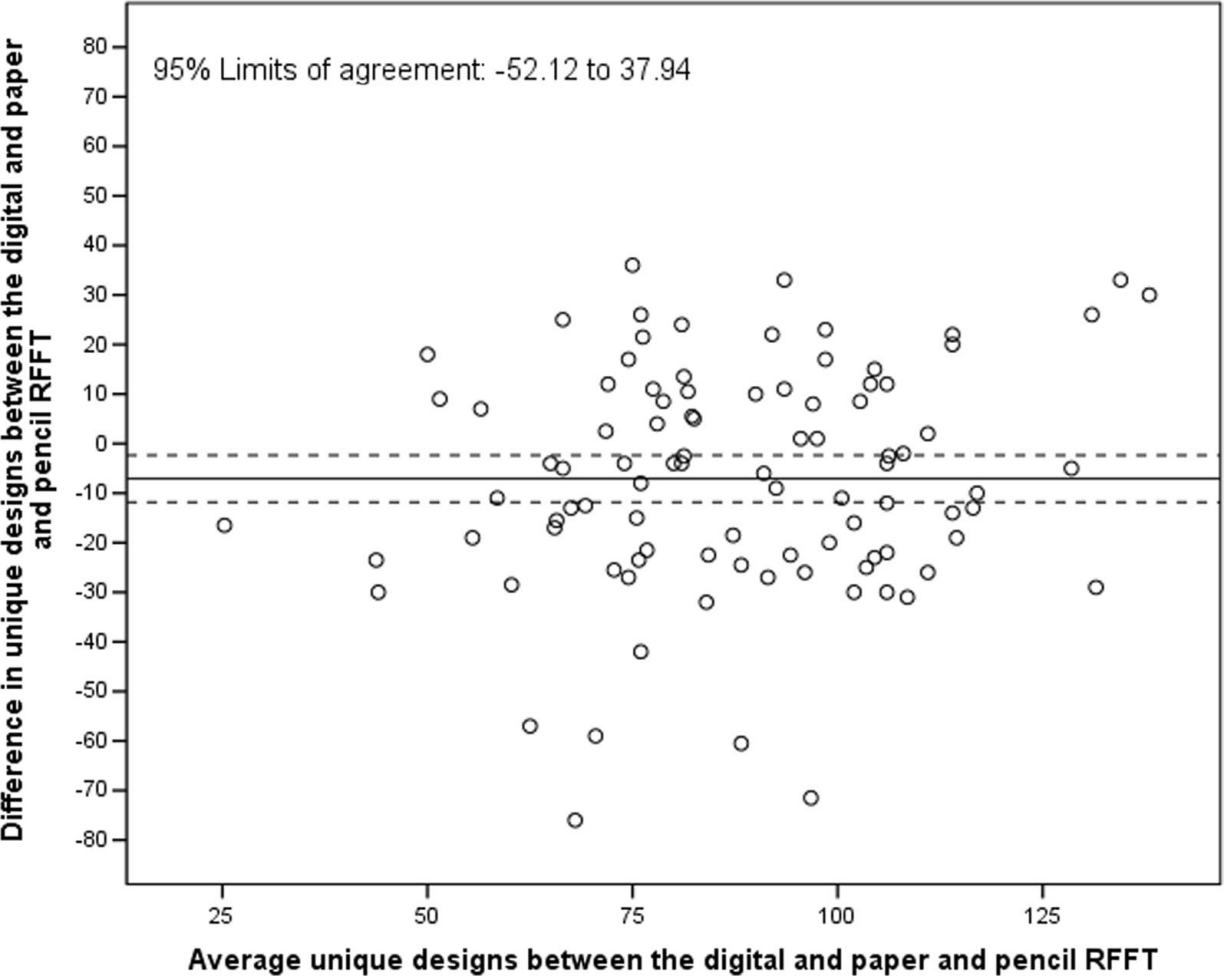
Bland–Altman plot of the digital RFFT (automatic scoring) and paper-and-pencil RFFT (number of unique designs)

For the number of unique designs, the ICC and LCCC between digital RFFT with automatic scoring and paper-and-pencil RFFT were 0.54 (95% CI 0.37, 0.67) and 0.60 (95% CI 0.43, 0.70), respectively. The number of unique designs on the digital RFFT with automatic scoring was significantly smaller than those of the paper-and-pencil RFFT (mean difference = − 7.09, 95% CI − 11.80, − 2.38; Fig. 5). The difference did not increase with a higher average number of unique designs on the digital RFFT and the paper-and-pencil RFFT. The 95% limits of agreement were − 52.12 and 37.94.

**Fig. 6:**
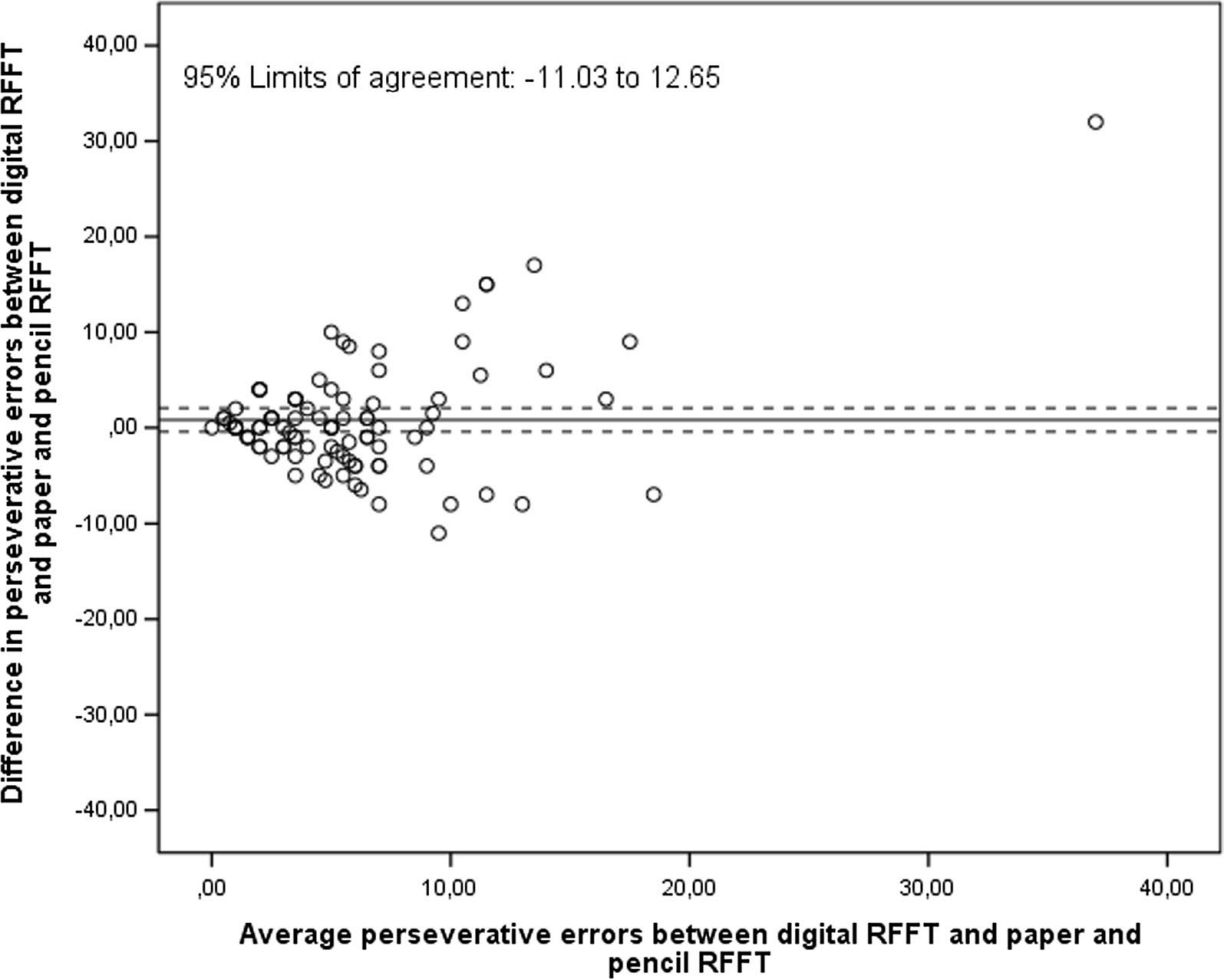
Bland–Altman plot of the digital RFFT (automatic scoring) and paper-and-pencil RFFT (number of perseverative errors)

For the number of perseverative errors, the ICC and LCCC between digital RFFT with automatic scoring and paper-and-pencil RFFT were 0.47 (95% CI 0.30, 0.62) and 0.44 (95% CI 0.24, 0.57), respectively. The number of perseverative errors on the digital RFFT did not differ significantly from those of the paper-and-pencil RFFT (mean difference = 0.81, 95% CI − 0.43, 2.05; Fig. 6). The 95% limits of agreement were − 11.03 and 12.65.

### Convergent validity: comparison digital RFFT with age and educational level

**Fig. 7:**
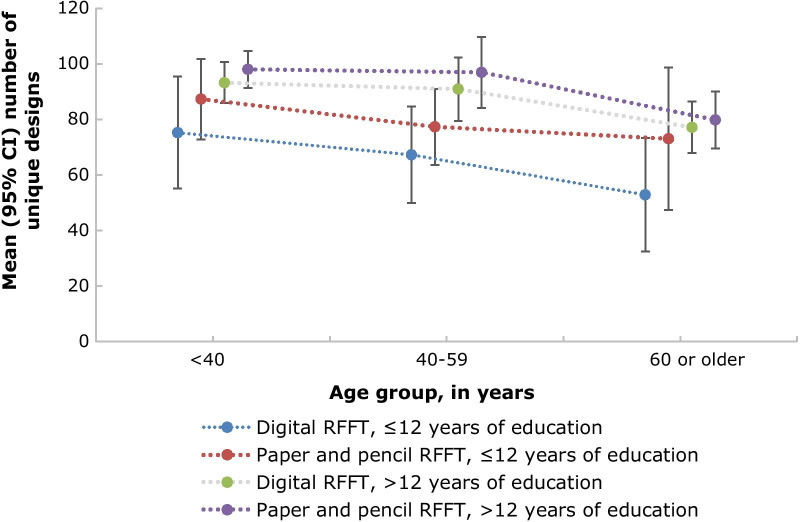
Mean (95% CI) of unique designs per age group and educational level

The mean (95% CI) number of unique designs per group of age and educational level are shown in Fig. 7. A higher number of unique designs on the digital RFFT was associated with higher educational level (Spearman’s *r* = 0.43, *p* < 0.001), and younger age (Spearman’s *r* = − 0.36, *p* < 0.001).

**Fig. 8:**
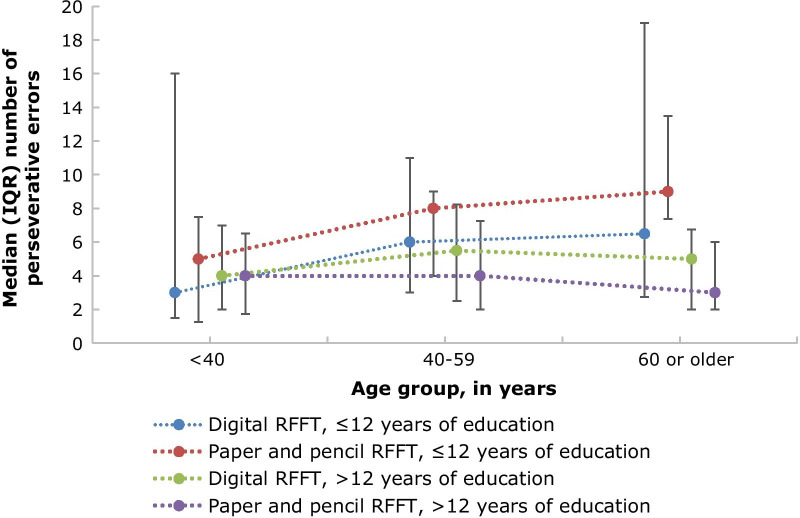
Median (IQR) of perseverative errors per age group and educational level

The median (IQR) number of perseverative errors was not associated with educational level (Spearman’s *r* = − 0.14, *p* = 0.19) and not with age (Spearman’s *r* = 0.20, *p* = 0.06) (Fig. 8).

### Test–retest reliability

Characteristics of the responders returning for the second visit are shown in Additional file 1: Appendix 3. The median (IQR) follow-up period was 7 (7–9.5) days. Females were more likely to respond than males (81.8% for females compared to 61.5% for males; *p* < 0.05; see Additional file 1: Appendix 4). In general, we also observed small but statistically non-significant differences between responders and non-responders in age, education, and number of unique designs, and number of perseverative errors on the digital RFFT during the first visit. Compared to non-responders, responders were younger (mean (SD) age: 39.7 (14.7) versus 43.2 (15.6) and more often highly educated (76.8% versus 68.0%). Furthermore, responders had a higher number of unique designs (mean (SD) 86.9 (25.1) versus 80.9 (29.2)) and higher number of perseverative errors (median (IQR): 5.0 (3.0–8.0) versus 4.0 (1.5–7.0)) than non-responders.

**Fig. 9:**
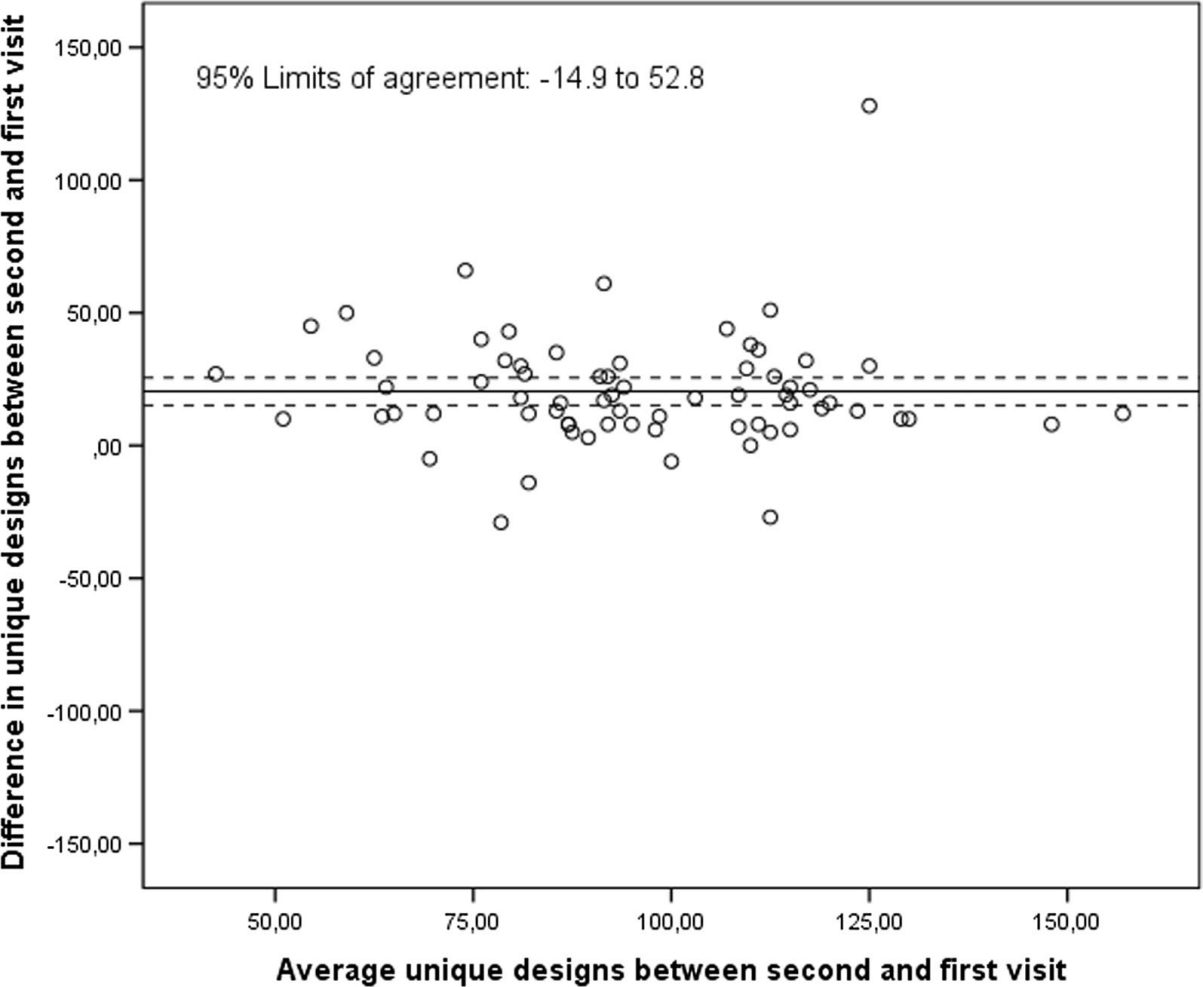
Bland–Altman plot comparing the first and second visit on the number of unique designs (digital RFFT)

For the number of unique designs on the digital RFFT with the automatic scoring, the ICC between the first and second visit was 0.57 (95% CI − 0.01, 0.81). The number of unique designs on the digital RFFT with automatic scoring during the second visit was significantly higher to those from the first visit (mean difference = 18.9, 95% CI 14.8, 23.1; Fig. 9). This difference did not increase with a higher average number of unique designs.

**Fig. 10:**
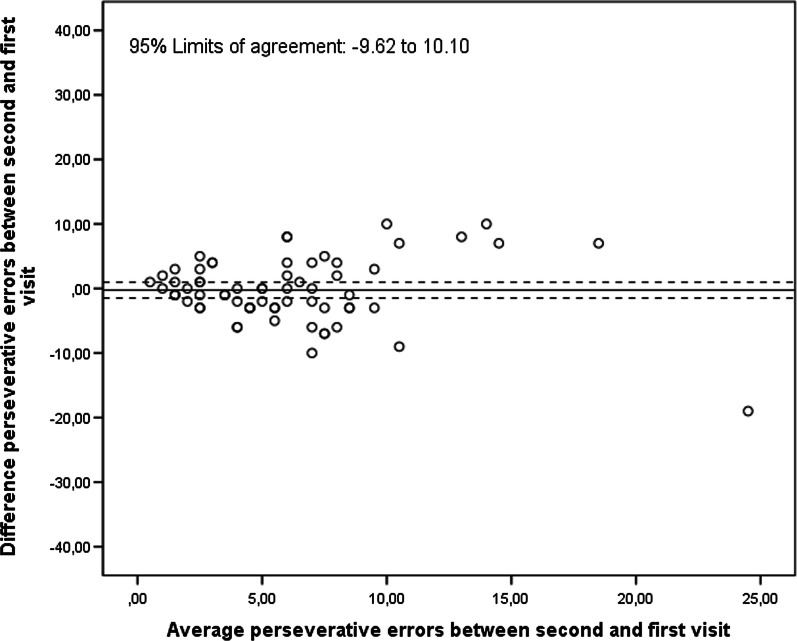
Bland–Altman plot comparing the first and second visit on the number of perseverative errors (digital RFFT)

For the number of perseverative errors on the digital RFFT with the automatic scoring, the ICC between the first and second visit was 0.48 (95% CI 0.27, 0.65). The number of perseverative errors on the digital RFFT with automatic scoring of the second visit did not differ significantly from those of the first visit (mean difference = − 0.24, 95% CI − 0.99, 1.48; Fig. 10). The 95% limits of agreement were − 9.62 and 10.10.

## Discussion

### Criterion validity

In the current study, we found that the automatic scoring of the newly developed digital RFFT has a good criterion validity. High ICCs were found between the scores provided by the automatic scoring of the digital RFFT and those from human raters. Moreover, excellent sensitivities and specificities were found for the automatic scoring of digital RFFT in both unique designs and perseverative errors. All together, these findings indicate good criterion validity, suggesting that the automatic scoring corresponds closely with the manual scoring of the digital RFFT.

The automatic scoring of the digital RFFT was slightly, but systematically lower in terms of the number of unique designs compared to the manual scoring of the digital RFFT. In other words, the automatic scoring of the digital RFFT may be stricter in granting unique designs than a manual scorer. Therefore, researchers should be cautious with using the digital RFFT and paper-and-pencil RFFT interchangeably. However, this difference was smaller than the difference between the two human raters. This demonstrates that minor differences in the interpretation of RFFT sheets are common between human raters but also between an algorithm and a human rater. Furthermore, because of the large variability in unique designs between participants, the digital RFFT could still discriminate people on their cognitive ability. For perseverative errors, the automatic scoring did not systematically differ from the manual scoring. So, although the digital RFFT has good psychometric properties, researchers should be cautious when comparing digital RFFT scores with paper-and-pencil RFFT scores.

The congruence between the digital RFFT (automatic scoring) and the paper-and-pencil RFFT (manual scoring) was moderate for unique designs and poor for perseverative errors, but may have been underestimated for several reasons. Firstly, the timing of the assessment could play a role. Participants immediately performed the paper-and-pencil RFFT after finishing the digital RFFT (or vice versa). We observed substantial learning effects between the first and second test at visit one which could be explained by this timing, and which could have weakened the ICC. Namely, participants starting with the digital RFFT with automatic scoring as a first test had a mean (SD) of 74.1 (24.4) unique designs. When these participants subsequently performed the paper-and-pencil RFFT as their second test, they had a mean (SD) of 96.9 (22.5) unique designs. So, substantial learning effects were present. Reversely, participants starting with the paper-and-pencil RFFT had a mean (SD) of 85.3 (20.8) unique designs. Later on, these participants had a mean (SD) of 96.5 (23.2) unique designs on the digital RFFT with automatic scoring. Again, these findings suggest that major learning effects occurred. Secondly, an early preliminary evaluation of the comprehensibility of the instructions of the digital RFFT indicated that the instructions of the digital RFFT may not have been sufficiently clear to all participants. Because of this issue, the first participants’ RFFT scores may have not been fully representative of their actual executive cognitive functioning. Ultimately, the congruence between digital RFFT (automatic scoring) and paper-and-pencil RFFT (manual scoring) may have been undermined by the timing of the assessments and initial issues with the instruction of the digital RFFT.

We found that participants had a systematically lower number of unique designs on the digital RFFT (automatic scoring) than on the paper-and-pencil RFFT (manual scoring). A potential explanation for this finding may be that some individuals were less familiar with using an iPad. In line with this, the difference in unique designs between digital and paper-and-pencil RFFT was most pronounced in elderly participants with less than 12 years educational background. Therefore, researchers should be cautious when administering the digital RFFT to individuals who are unfamiliar with using an iPad.

### Convergent validity

For convergent validity of the digital RFFT, we found that the number of unique designs discriminates between different levels of education and age. Participants from higher educational level scored more unique designs on the digital RFFT than less educated participants, and participants from younger age scored more unique designs on the digital RFFT than participants from older age. The number of perseverative errors was not associated with educational level nor with age. Still, overall, younger and highly educated individuals performed better than older individuals, as they had more unique designs relative to the number of perseverative errors. These results are in line with previous studies, which found that younger individuals and highly educated individuals performed better on the RFFT in terms of unique designs, but not in terms of perseverative errors [9, 12]. A potential explanation for this discrepancy may be that people do not differ much in their number of perseverative errors, and that our study might be too small to detect a difference. Put together, individuals who are younger and more highly educated performed better on the digital RFFT.

### Test–retest reliability

For unique designs, the test–retest reliability of the digital RFFT between visit one and visit two was moderate. Again, this may be explained by learning effects as the period between the two visits was approximately 1–2 weeks. The mean (sd) number of unique designs on the digital RFFT was 84.2 (26.0) and 104.4 (22.7) for the first and second visit, respectively. When replacing the ICC (absolute) by ICC (consistency), which is not affected by learning effects, the ICC improved from 0.57 (95% CI − 0.01, 0.81) to 0.74 (95% CI 0.61, 0.83). These points highlight that learning effects may have substantially impacted test–retest reliability.

### Strengths and limitations

Strengths of this study include the broad and detailed assessment of psychometric properties of the digital RFFT. We compared the automatic scoring of the digital RFFT with the manual scoring of the digital and the paper-and-pencil RFFT. To examine the validity of the automatic scoring of the digital RFFT, we specifically investigated sensitivity and specificity of identifying an individual box as a unique design or perseverative error compared to human assessment, rather than only comparing total numbers of unique designs and perseverative errors. Also, we investigated the test–retest reliability of the digital RFFT, and compared the RFFT performance in relation to relevant socio-demographic characteristics such as age and educational level. Thus, a wide range of psychometric properties of the digital RFFT was investigated in this study.

The low number of individuals aged 60 years and older and less educated people included is an important limitation in the current study. The recruitment method partly occurred online, and could therefore have made it more difficult for individuals aged 60 years and older and individuals from low educational level to enrol in this study. Therefore, our results on the validity and reliability of the digital RFFT might not be fully generalizable to these subgroups. Nevertheless, when comparing our paper-and-pencil RFFT scores (among participants who started with this test) with those of a population-based sample of Kuiper et al. [19], we found similar scores for the mean number of unique designs (85.3(20.8) and 85.2(24.4), respectively). In addition, we did not screen our participants for psychiatric disorders or cognitive impairment. However, we judge the risk of such individuals entering in this study as low as individuals had to make an online appointment on their own initiative and had to come to the research site. Another limitation of this study was that the short period between various RFFT performances in the study design may have introduced learning effects. Participants consecutively performed two RFFT tests during the first visit (digital RFFT and paper-and-pencil RFFT) and performed the digital RFFT again at a second visit after 1 week. Over the series of RFFT tests, participants may have refined their strategy, resulting in improved performance in subsequent RFFT tests.

### Implications

Results of the current study suggest that the digital RFFT is a valid and reliable instrument to measure executive cognitive function and is a feasible alternative to the paper-and-pencil RFFT in large-scale cohort studies. Still, due to systematic differences between the digital and paper-and-pencil RFFT, the scores between these two tests cannot be used interchangeably. Participants can perform the digital RFFT independently on an iPad, making it less labour-intensive to conduct the RFFT. Also, the assessment of the digital RFFT is less labour-intensive and time-consuming than the paper-and-pencil RFFT, as RFFT patterns are automatically stored and processed into unique designs and perseverative errors. This is in sharp contrast with manually assessing the RFFT, which took approximately 15 min per participant in this study. Furthermore, the automatic scoring of the performance on the RFFT is not sensitive for inter-rater differences. This allows for further large-scale investigations into the pathways of cognitive decline in the population. Due to initial issues with clarity of instructions of the digital RFFT, we provided specific recommendations for improved instructions to improve its validity and reliability (see Additional file 1: Appendix 5). These recommendations have been incorporated in the final version of the digital RFFT. This final version of the digital RFFT has been implemented in the third screening round of Lifelines as they were seeking a more efficient alternative cognitive assessment in their large-scale cohort study (www.lifelines.nl) [19]. Our recommendations included instructions to watch example videos on the iPad prior to performing the RFFT, the use of a separate instruction card next to the iPad, and instructions on not to skip practice sheets before starting with the actual digital RFFT sheets. We also recommended to add more simplified examples of designs connecting two dots with a straight line. Namely, the goal of the RFFT is to connect minimally two dots with a straight line. Ultimately, the digital RFFT appears to be valid and reliable in assessing executive cognitive functioning, and can be incorporated in large-scale cohort studies.

### Recommendations for future research

Future studies should investigate the validity of the digital RFFT in a large sample with sufficient older individuals and individuals with a low educational level. Moreover, a translation and cross-cultural validation of the digital RFFT into other languages would enhance a more widespread use of the digital RFFT in other countries. Finally, the responsiveness of the digital RFFT should be validated.

## Conclusions

The automatic scoring of the digital RFFT has excellent criterion validity and the number of unique designs discriminates between levels of education and age. However, learning effects may have weakened agreement with the paper-and-pencil RFFT and the second digital RFFT (test–retest reliability). We provide specific recommendations to clarify the instructions. Besides, the digital RFFT does not require human effort in the assessment. Therefore, the digital RFFT is a valid and reliable instrument to measure executive cognitive function among the general population and can be used as an alternative to the paper-and-pencil RFFT in large-scale cohort studies, but its scores cannot be compared directly to the paper-and-pencil RFFT.

## Supplementary Information

**Additional file 1**. Definitions of educational levels, criteria to identify erroneous designs, characteristics of the study population that participated in the second visit, characteristics of the responders and non-responders and the list of improvements to provide clearer instructions for the digital RFFT.

## Data Availability

The Dutch digital RFFT and datasets analysed during the current study are available from the corresponding author upon reasonable request.

## Abbreviations

RFFT: Ruff Figural Fluency Test
SD: Standard deviation
ICC: Intraclass correlation coefficient
LCCC: Lin’s concordance correlation coefficient
IQR: Inter quartile range
UD: Unique designs
PE: Perseverative errors
CI: Confidence interval
