# Supplementary material for: The validity and reliability of a digital Ruff Figural Fluency Test (RFFT)
Source: BMC Psychol. 2021 Apr 28;9:65. doi: 10.1186/s40359-021-00566-x (PMC8080381; doi:10.1186/s40359-021-00566-x)
Supplement: Supplementary file 1 — Additional file 1. Definitions of educational levels, criteria to identify erroneous designs, characteristics of the study population that participated in the second visit, characteristics of the responders and non-responders and the list of improvements to provide clearer instructions for the digital RFFT. [file 40359_2021_566_MOESM1_ESM.docx]

**Title**

The validity and reliability of a digital Ruff Figural Fluency Test (RFFT).

**Authors**

Vrijsen J^1#^*, van Erpecum CP^1#^, de Rooij SE^2^, Niebuur J^1^, Smidt N^1^

^1^ University of Groningen, University Medical Centre Groningen, Department of Epidemiology, Groningen, the Netherlands

^2^ Medical Spectrum Twente, Medical School, Enschede, the Netherlands

^#^Contributed equally

*Corresponding author. University Medical Centre Groningen, Department of Epidemiology, Hanzeplein 1, PO Box 30 001, FA40, 9700 RB Groningen, the Netherlands. E-mail: j.vrijsen@umcg.nl **Appendix 1:** Definitions of low, middle, and high level of education based on the International

Standard Classification of Education.

| Highest level of completed education | Definition |
| --- | --- |
| Low | Less than primary education Primary education Lower secondary education |
| Middle | Upper secondary education Post-secondary non-tertiary education |
| High | Short-cycle tertiary education Bachelor or equivalent education Master or equivalent education Doctoral or equivalent education |

**Appendix 2:** Criteria to identify for erroneous designs iPad

1) Widow: when drawing designs, participants should draw lines starting and ending within a dot. If a drawed line is starting or ending too far away from a dot, the drawing will be considered as a ‘widow’ and counted as an erroneous design. How far away from a dot is a line allowed to start or end?

The line should end within one radius distance from the dot (see image below). Yet, a square is used (instead of a circle) to determine the maximum distance. As a result, the line may end further away from the dot if it ends at the corner of the square (i.e. left-upper corner, right-upper corner, left-bottom corner, right-bottom corner).

r: radius of the dot

r

r

r

*Example*


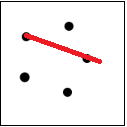

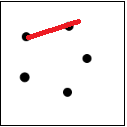


**Correct Incorrect**

The line may start/end further
away from the dot if in the left-upper,
right-upper, left-bottom or right-bottom
side from the dot.

2) Bent line
Drawings containing bent lines are counted as erroneous designs. To determine whether a line is bent too much, Pythagoras’ law is used.

*Example*


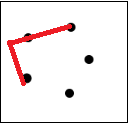

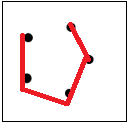


**Correct Incorrect**

The line between the two

dots at the bottom contains
two bends that are both counted as
correct.

3) Double line
Lines drawn double (i.e. two lines drawn between the same pair of dots) are identified and counted as erroneous designs.

*Example*


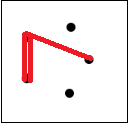

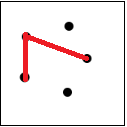


**Correct Incorrect**


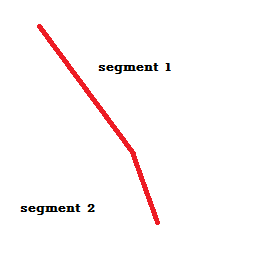
4) Segments unintended to be a line
A line is seen as an actual line if:
- a line is long enough (e.g., a dot drawn is not long enough)
- the line only contains at least two segments
- the line starts within a dot and ends within a dot

*Example*


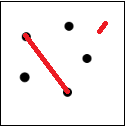

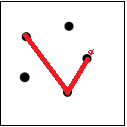


**Correct Incorrect**

Unintended line starts at
dot and ends at dot, and is
therefore not counted as
erroneous design.

**Appendix 3:** Characteristics of the study population (N=69) that participated in the second visit*.

|  | **Total study population (N=69)** | **Allocation to 1^st^ RFFT** | |  |
| --- | --- | --- | --- | --- |
|  |  | **Digital (N=37)** | **Paper-and-pencil (N=32)** | **P-value** |
| Sex (female) | *45* (65.2) | *22* (59.5) | *23* (71.9) | 0.28 |
| Age in years, mean (SD) | *39.7* (14.7) | *41.7* (16.3) | *37.3* (12.6) | 0.21 |
| Age categories |  |  |  | 0.70 |
| <40 years | *43* (62.3) | *22* (59.5) | *21* (65.6) |  |
| 40-59 years | *17* (24.6) | *9* (24.3) | *8* (25.0) |  |
| ≥60 years | *9* (13.0) | *6* (16.2) | *3* (9.4) |  |
| Level of education |  |  |  | 0.67 |
| Low | *7* (10.1) | *4* (10.8) | *3* (9.4) |  |
| Middle | *9* (13.0) | *6* (16.2) | *3* (9.4) |  |
| High | *53* (76.8) | *27* (73.0) | *26* (81.3) |  |
| Years of education |  |  |  | 0.22 |
| ≤12 years education | *18* (26.1) | *12* (32.4) | *6* (18.8) |  |
| >12 years education | *51* (73.9) | *25* (67.6) | *26* (81.3) |  |
| *Digital RFFT (First visit)* |  |  |  |  |
| UD (automatic), mean(sd) | 85.5 (25.3) | 75.8 (24.0) | 96.7 (22.2) | 0.00 |
| PE (automatic), median (IQR) | 5.0 (3.0 – 8.0) | 4.0 (2.0-9.5) | 5.0 (3.0 -6.5) | 0.59 |
| UD (manual), mean(sd) | 86.9 (25.1) | 77.5 (24.2) | 97.8 (21.5) | 0.00 |
| PE (manual), median (IQR) | 5.0 (2.0-8.3) | 5.0 (2.0 – 8.8) | 5.0 (2.0-7.3) | 0.57 |
| *Digital RFFT (Second visit)* |  |  |  |  |
| UD (automatic), mean (SD) | 104.4 (22.7) | 102.5 (22.4) | 106.6 (23.2) | 0.46 |
| PE (automatic), median (IQR) | 6 (2 – 8.25) | 6 (2 – 7.5) | 6 (4 – 9) | 0.57 |

SD: standard deviation. **N* (%) is presented unless indicated otherwise.

**Appendix 4:** Characteristics of the responders and non-responders during the second visit. *N* (%) is presented unless indicated otherwise.

|  | Responders  (n=69) | | Non-responders (n=25) | | P-value |
| --- | --- | --- | --- | --- | --- |
| Sex (female) | *45* | (65.2) | *10* | (40.0) | 0.03 |
| Age in years, mean (sd) | *39.7* | (14.7) | 43.2 | (15.6) | 0.31 |
| Age categories |  |  |  |  | 0.45 |
| <40 years | *43* | (62.3) | *12* | (48.0) |  |
| 40-59 years | *17* | (24.6) | *8* | (32.0) |  |
| ≥60 years | *9* | (13.0) | *5* | (20.0) |  |
| Level of education |  |  |  |  | 0.66 |
| Low | *7* | (10.1) | *4* | (16.0) |  |
| Middle | *9* | (13.0) | *4* | (16.0) |  |
| High | *53* | (76.8) | *17* | (68.0) |  |
| Years of education |  |  |  |  | 0.57 |
| ≤12 years education | *18* | (26.1) | *8* | (32.0) |  |
| >12 years education | *51* | (73.9) | *17* | (68.0) |  |
| Digital RFFT (first visit) |  |  |  |  |  |
| UD (automatic), mean(sd) | *86.9* | (25.1) | *80.9* | (29.2) | 0.42 |
| PE (automatic), median(IQR) | *5.0* | (3.0-8.0) | *4.0* | (1.5-7.0) | 0.51 |

**Appendix 5:** List of improvements to provide clearer instruction for the digital RFFT

|  | **Current study** | **Recommendations** |
| --- | --- | --- |
| **Example video** |  |  |
| Task to watch example video | Optional | Mandatory |
| Examples in example video | Mainly complex examples (e.g. a line connecting all dots) | Start with simple examples (e.g. a line connecting two dots) |
| Timing of showing an example during instructions | A complex example is shown when it is instructed to connect at least two dots with a straight line | The examples should be shown after it is instructed to connect at least two dots with a straight line |
| **Configurations of digital RFFT** |  |  |
| Scoring criteria | A square was used to determine the maximum distance from the dot. As a result, the line may end further away from the dot if it ends at the corner of the square. | A circle should be used to determine the maximum distance. |
| Double lines | Two lines drawn between the same pair of dots are identified and counted as erroneous designs. | Two lines drawn between the same pair of dots are **not** counted as erroneous designs. |
| Unintended lines | Designs including unintended lines are counted as erroneous designs. | Designs including unintended lines are not counted as erroneous designs. |
